# Bioinspired Bone Seed 3D‐Printed Scaffold via Trapping Black Phosphorus Nanosheet for Bone Regeneration

**DOI:** 10.1002/smsc.202300357

**Published:** 2024-05-11

**Authors:** Zhengwei Cai, Zhijie Chen, Yuan Tang, Liang Cheng, Minglong Qiu, Ningtao Wang, Wei Jiang, Zhanchun Li, Catarina Leite Pereira, Yunhai Zhang, Bruno Sarmento, Wenguo Cui

**Affiliations:** ^1^ Department of Orthopaedics Shanghai Key Laboratory for Prevention and Treatment of Bone and Joint Diseases Shanghai Institute of Traumatology and Orthopaedics Ruijin Hospital Shanghai Jiao Tong University School of Medicine 197 Ruijin 2nd Road Shanghai 200025 P. R. China; ^2^ Department of Orthopaedic Surgery Renji Hospital, School of Medicine Shanghai Jiao Tong University Shanghai 200127 P. R. China; ^3^ Department of Orthopedics The First Affiliated Hospital of Anhui Medical University 218 Jixi Road Shushan District Hefei Anhui 230022 P. R. China; ^4^ I3‐Instituto de Investigação e Inovação Em Saúde and INEB‐Instituto de Engenharia Biomédica Universidade Do Porto Rua Alfredo Allen 208 4200‐135 Porto Portugal; ^5^ Department of Orthopedics Wuxi Branch of Ruijin Hospital Wuxi Jiangsu 214106 P. R. China; ^6^ IUCS‐Instituto Universitário de Ciências da Saúde CESPU Rua Central de Gandra 1317 4585‐116 Gandra Portugal

**Keywords:** black phosphorus, bone regenerations, bone seeds, endogenous mineralizations, 3D scaffolds

## Abstract

Significance of endogenous mineralization in bone reconstruction is paramount, as it facilitates the accumulation of calcium ions for bone tissue deposition. However, conventional 3D‐printed scaffolds lack the capacity for calcium ion enrichment and mineralization, coupled with their low bone inductive activity. Inspired from the development of natural plant seeds, a biomimetic 3D printing scaffold is developed by implanting photosensitive black phosphorus “bone seeds” (BS), guiding a sequential process mirroring rooting (osteoblast recruitment), sprouting (fibrous callus mineralization), flowering (osseous callus formation), and fruiting (callus plasticity). BS were trapped onto porous 3D polycaprolactone (PCL) scaffolds with aminated surfaces *via* electrostatic interactions between phosphates and amino groups, creating the PCL‐BS scaffold that can actively capture calcium ions for accelerating the endogenous regeneration of critical bone defects. In vitro and in vivo experiments show that the PCL‐BS scaffold has good biocompatibility and strong osteogenic ability for rapid new bone regeneration under near‐infrared (NIR) stimulation. In addition, whole transcriptome sequencing analysis is performed to reveal the transcriptomic mechanism of BS involved in signal transduction and network regulation during bone regeneration. This NIR light‐regulated biomimetic BS inspired by seed planting, introduces a pioneering concept in the design of 3D printing bone repair scaffolds.

## Graphical abstract

Inspired by natural plant seeds, a biomimetic 3D‐printed scaffold is designed for the trapping of photosensitive black phosphorus “bone seeds (BS)” via electrostatic interactions that can actively capture calcium ions to accelerate endogenous bone regeneration. Furthermore, whole transcriptome sequencing analysis is performed to reveal the transcriptomic mechanism of “BS” involved in signal transduction and network regulation.

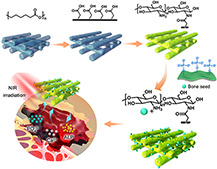

## 1 Introduction

Endogenous regeneration in bone is pivotal, divided into four stages: the hematoma phase, fibrous/cartilaginous callus phase, osseous callus phase, and callus remodeling phase, crucially involving the recruitment, mineralization, and crystallization of calcium ions, ultimately forming regenerative bone tissue.^[^ ^1^ ^]^ However, conventional bone repair strategies predominantly rely on the use of 3D‐printed scaffolds, which, although providing structural support and facilitating favorable 3D conditions for bone regeneration, exhibit limitations such as the inability to effectively enrich calcium ions, lack of mineralization capacity, and restricted clinical applicability due to their low bone inductive activity.^[^ ^2^, ^3^, ^4^ ^]^ To address this issue, a new strategy inspired by the life cycle of plant seeds is proposed: seeding bioinspired “bone seeds (BS)” on 3D scaffolds, aiming to analogize the stages of rooting (recruitment of osteogenic cells), germination (mineralization of fibrous healing tissue), flowering (formation of osseous regenerative tissue), and fruition (enhanced plasticity of healing tissue). Hence, this innovative biomimetic concept of “BS”, inspired by the natural growth process of plant seeds, holds the promise of overcoming the limitations of conventional 3D scaffolds by enhancing bone mineralization and deposition capabilities.

A proficient “BS” necessitates dual attributes: resemblance to natural bone tissue and robust osteogenic efficacy. Hydroxyapatite (HA), prevalent in natural bone, exhibits commendable biocompatibility.^[^ ^5^, ^6^ ^]^ Nevertheless, HA encounters formidable challenges—resistance to degradation, inadequate cell adhesion, and the absence of conducive 3D micro‐/nanostructures essential for proficient bone growth.^[^ ^7^, ^8^, ^9^ ^]^ These inherent limitations curtail HA's candidacy as a promising “BS.” Conversely, black phosphorus (BP), a novel 2D nonmetallic nanomaterial and phosphorus's most stable allotrope, emerges as a compelling contender for an “ideal BS.”^[^ ^10^, ^11^, ^12^ ^]^ Notably, BP mirrors crucial inorganic constituents of natural bone, aligning with its structural likeness. Moreover, BP boasts favorable attributes, encompassing low toxicity, degradability, and noteworthy osteogenic prowess, offering a promising avenue for enhanced bone regeneration.^[^ ^13^, ^14^, ^15^ ^]^ For instance, Gu and co‐workers reported that BP‐based hydrogel scaffolds can continuously degrade and supply phosphate to bind calcium ions to promote osseointegration.^[^ ^16^ ^]^ However, the phosphate produced by the degradation of BP degradation is easily lost to surrounding body fluids, forming ectopic mineralized crystals, which greatly increase the risk of ectopic bone formation. Consequently, beyond serving as a phosphorus source, BP‐based “BS” must also enhance their ability to recruit calcium ions, foster the formation of endogenous mineralized crystals, and stimulate in situ bone development.

To facilitate efficient calcium ion capture, insights and inspiration are drawn from the critical role of light sources in the developmental phases of natural plant growth. Photothermal therapy (PTT) is a commonly used method in tumor treatment, which can ablate and kill cancer cells by locally generating instantaneous high‐energy thermal effects under near‐infrared (NIR) irradiation by nanomaterials.^[^ ^17^, ^18^, ^19^, ^20^ ^]^ Nevertheless, photothermal microstimulation has been found to have a remarkable ability to activate cells expressing crucial factors such as alkaline phosphatase (ALP), heat shock protein (HSP), and other essential elements, leading to bone regeneration. For instance, Zhang et al.^[^ ^21^ ^]^ reported that NIR‐activated porous AuPd nanoparticles generate mild local heat, which helps to promote osteogenesis; Wang et al.^[^ ^22^ ^]^ found that NIR‐irradiated BP‐SrCl_2_/PLGA microspheres facilitated bone regeneration of femoral defects in rats. Although PTT is widely used in bone repair, it is still subject to the following limitations:^[^ ^23^, ^24^, ^25^ ^]^ 1) generation of nondegradable or highly toxic degradation products (e.g., heavy metal ions that cannot be metabolized in the human body) within conventional PTT nanomaterials such as metal–organic framework (MOF) materials, gold nanoparticles, and transition metal dichalcogenides; and 2) lack of physical structural support to simulate the natural bone trabecular structure.

**Scheme 1:**
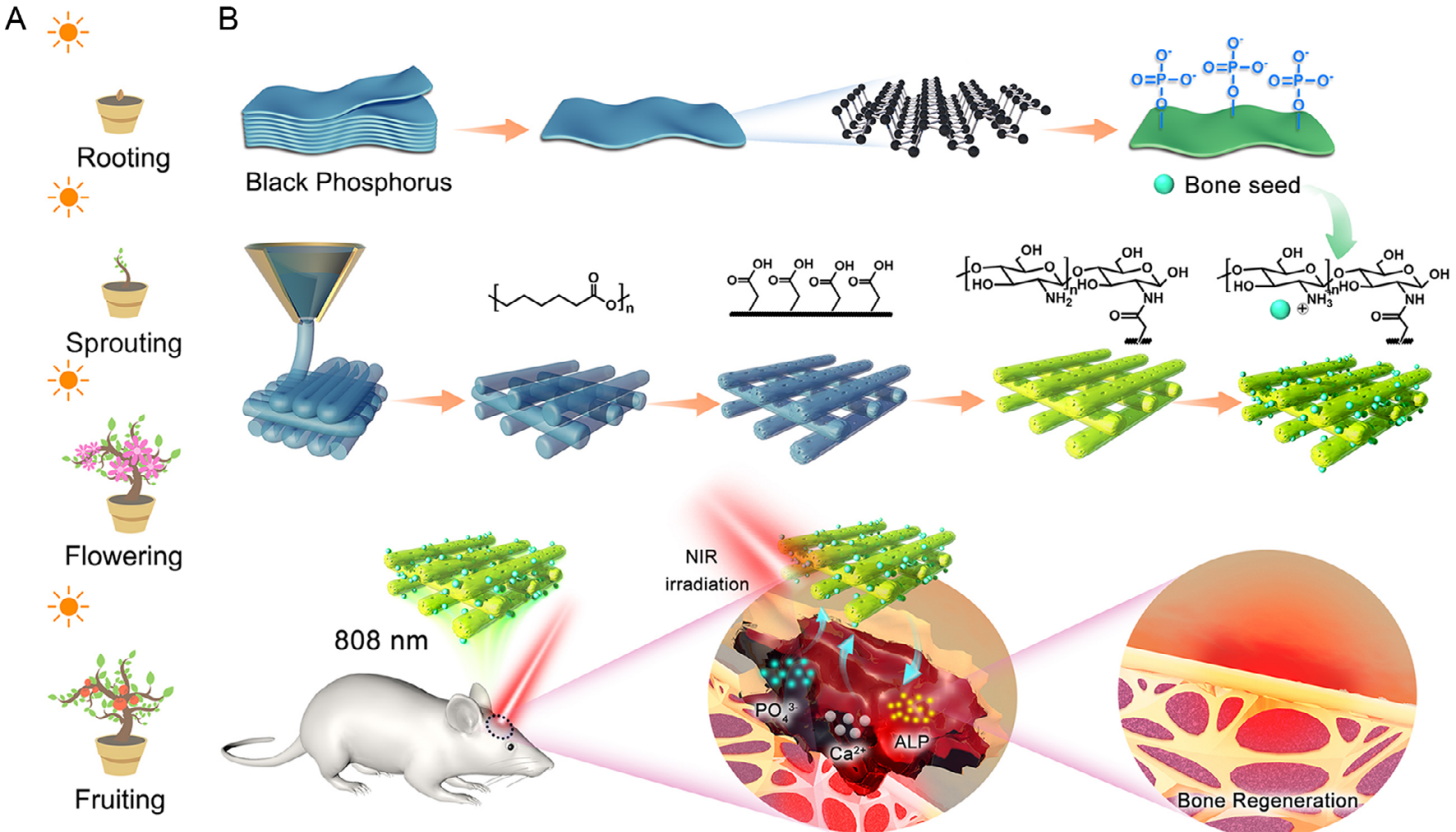
Illustration of photosensitive biomimetic “BS” mimicking the development of natural plant seeds. A) Natural seed growth process. B) The preparation process of bioinspired BS 3D‐printed scaffold, and the in situ deposition of calcium phosphate on the scaffold surface was promoted by 808 nm NIR light stimulation to accelerate bone regeneration.

Inspired by the development of natural plant seeds, a biomimetic 3D‐printed scaffold was designed for the trapping of photosensitive BP “BS” that can actively capture calcium ions to accelerate endogenous regeneration of critical bone defects. The process began with the preparation of BP nanosheets through a liquid‐phase stripping method, followed by the mild oxidation of their surfaces under weakly alkaline conditions; then, these “BS” were meticulously sown onto the surface of a porous 3D polycaprolactone (PCL) scaffold via electrostatic interactions between phosphates and amino groups. Under the influence of 808 nm NIR light stimulation, this approach facilitated the in situ deposition of calcium phosphate on the scaffold's surface (**Scheme** **1**). Concurrently, microthermal stimulation resulted in the upregulation of key osteogenesis‐related proteins promoting the repair of critical bone defects. This innovative approach presents a twofold advantage. On the one hand, local NIR heat stimulation helps to stimulate the growth process of natural “seeds” and accelerate the regeneration of bone tissue. On the other hand, simple modifications of traditional 3D‐printed scaffolds not only maintain the mechanical requirements of the bone but also better match the bone repair process. Furthermore, full transcriptome sequencing (RNA seq) analysis was performed to uncover the transcriptome mechanism of “BS” involvement in regulating signal transduction and network regulation in bone regeneration. This photoregulated bionic “BS” inspired by plant seeds is expected to open up a whole new concept for the design of 3D‐printed bone repair scaffolds.

## 2 Results and Discussion

### 2.1 Characterization of Black Phosphorus BS

**Figure 1:**
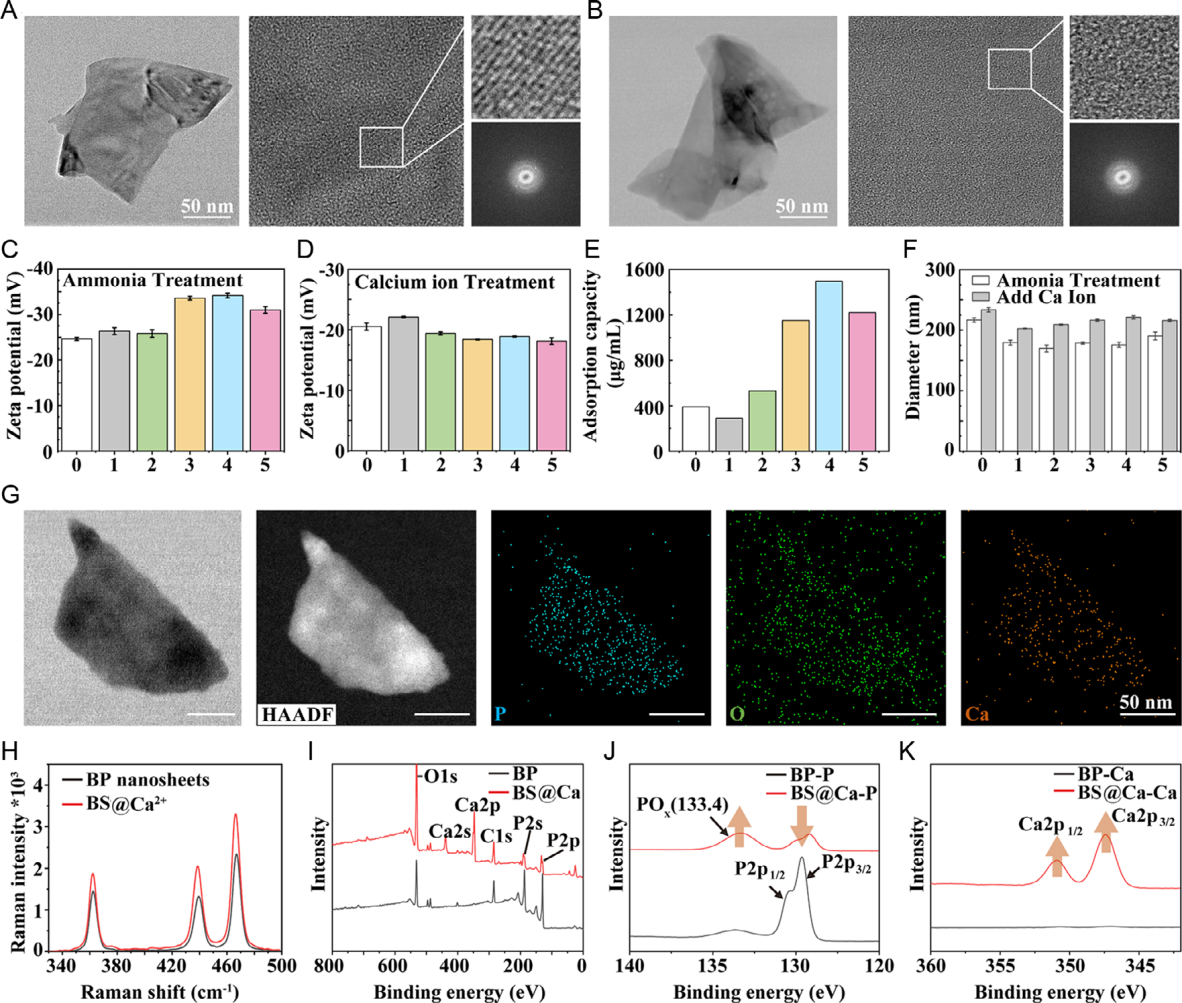
The preparation and characterization of “BS”. A,B) TEM and HRTEM nanolattice images of bare BP and BS@Ca, adsorbed with Ca^2+^. C,D) Zeta potential of BP after different alkalinization concentrations (0, 1, 5, 10, 15, 25 mM) and after Ca^2+^ adsorption. E,F) DLS before and after Ca^2+^ adsorption and actual amount of Ca^2+^ adsorbed. G) HRTEM images of BS@Ca in bright‐field and dark‐field modes, respectively, and its elemental scan mapping. H) Raman spectra of BP and BS@Ca. I–K) XPS spectra of BP and BS@Ca.

To augment the negative charge and calcium ion‐trapping efficacy of BP nanosheets, a mild weak base ammonia was used to oxidize the BP surface. First, the BP was exfoliated from BP bulks by liquid exfoliation in anhydrous *n*‐methyl‐2‐pyrrolidone (NMP) solution, which was added in ammonium hydroxide solution and stirred at 40 °C for 2 h to generate BP BS. The 2D surface morphologies of BP and BS were identified using high‐resolution transmission electron microscopy (HRTEM) (**Figure** **1**A,B); both BP and BS showed a uniform 2D sheet. However, upon treatment ammonia, the HRTEM image of BS revealed an absence of lattice fringes or diffraction spots compared to bare BPs (Figure 1B), signifying successful BP surface oxidation. In addition, to explore the optimal concentration of ammonia treatment, dynamic light scattering (DLS) and zeta potential were used to monitor BP before and after ammonia treatment (Figure 1C,F). At 15 mM ammonia concentration, a reduction in BP particle size from 216.80 ± 3.75 to 175.73 ± 4.23 nm and a decrease in zeta potential from −24.63 ± 0.33 to −34.16 ± 0.52 mV were observed. It is clearly shown that weak alkali treatment can effectively increase the negative charge on the surface of BS.

Furthermore, to verify the calcium ion‐capturing ability of BS, the CaCl_2_ solution was introduced to the BS solution and incubated in the dark. Upon calcium trapping on BS (BS@Ca), a substantial increase in both average particle size and zeta potential was noted. Among them, the particle size of BS treated with 15 mM ammonia increased to 211.2 ± 2.81 nm, and the zeta potential increased to −18.9 ± 0.08 mV (Figure 1D,F). Subsequently, a calcium assay kit was used to evaluate the calcium ion‐trapping efficiency of various BS samples. Similarly, we found that Ca^2+^ trapping ability of 15 mM ammonia treatment was the most significant. Compared with BP, the trapping ability increased by 3.8 times to 1.50 ± 0.04 mg mL^−1^ (Figure 1E). In addition, through HRTEM and energy dispersive spectroscopy (EDS) elemental mapping (Figure 1G), it was found that the 2D surface of BS@Ca was much rougher and there was obvious Ca^2+^ deposition. The Raman scattering spectrum showed BS@Ca with slight redshifts (Figure 1H). Moreover, the composition of BS@Ca was examined using X‐ray photoelectron spectroscopy (XPS). In Figure 1I–K, the typical P2p1/2 and P2p3/2 doublet peaks of BP crystals were significantly reduced, but at 133.4 eV it exhibits typical characteristic peaks of oxidized phosphorus, which prove the existence of phosphate anions on the surface of BS. Furthermore, characteristic peaks of Ca2p1/2 and Ca2p3/2 at 347.6 and 352.2 eV confirmed the active capture of Ca^2+^ by BS. Therefore, the BS obtained by weak alkali treatment have better Ca^2+^ capturing ability than traditional BP nanosheets.

### 2.2 Fabrication and Characterization of BS‐Sowed PCL 3D Printing Scaffold

Following the confirmation of BP BS capability to entrap calcium ions and facilitate the in situ mineralization of BP nanosheets, we recognized the advancements in 3D additive technologies, offering personalized solutions for diverse tissue engineering requirements aligned with varying physiological conditions. Nonetheless, current 3D printing predominantly emphasizes replicating the physical attributes of natural tissues, focusing on mechanics, spatial structure, and porosity, while an in‐depth understanding and replication of the distinct physiological microenvironment of diverse tissues remain underexplored.^[^ ^26^, ^27^, ^28^ ^]^ Therefore, we constructed 3D PCL porous scaffolds using a melt extrusion 3D printer with 25 G injection needle to serve as a base template. To ensure efficient loading of BS onto the scaffolds, porous carboxylate PCL scaffolds (PCL‐COOH) were obtained by alkalinization with 5 M sodium hydroxide, followed by covalent grafting of chitosan using the coupling reaction of carboxy amino groups and effective loading of BSs using the electrostatic interaction between the positive charge amino group of chitosan groups and the negative charge on the surface of oxidized BP nanosheets.

**Figure 2:**
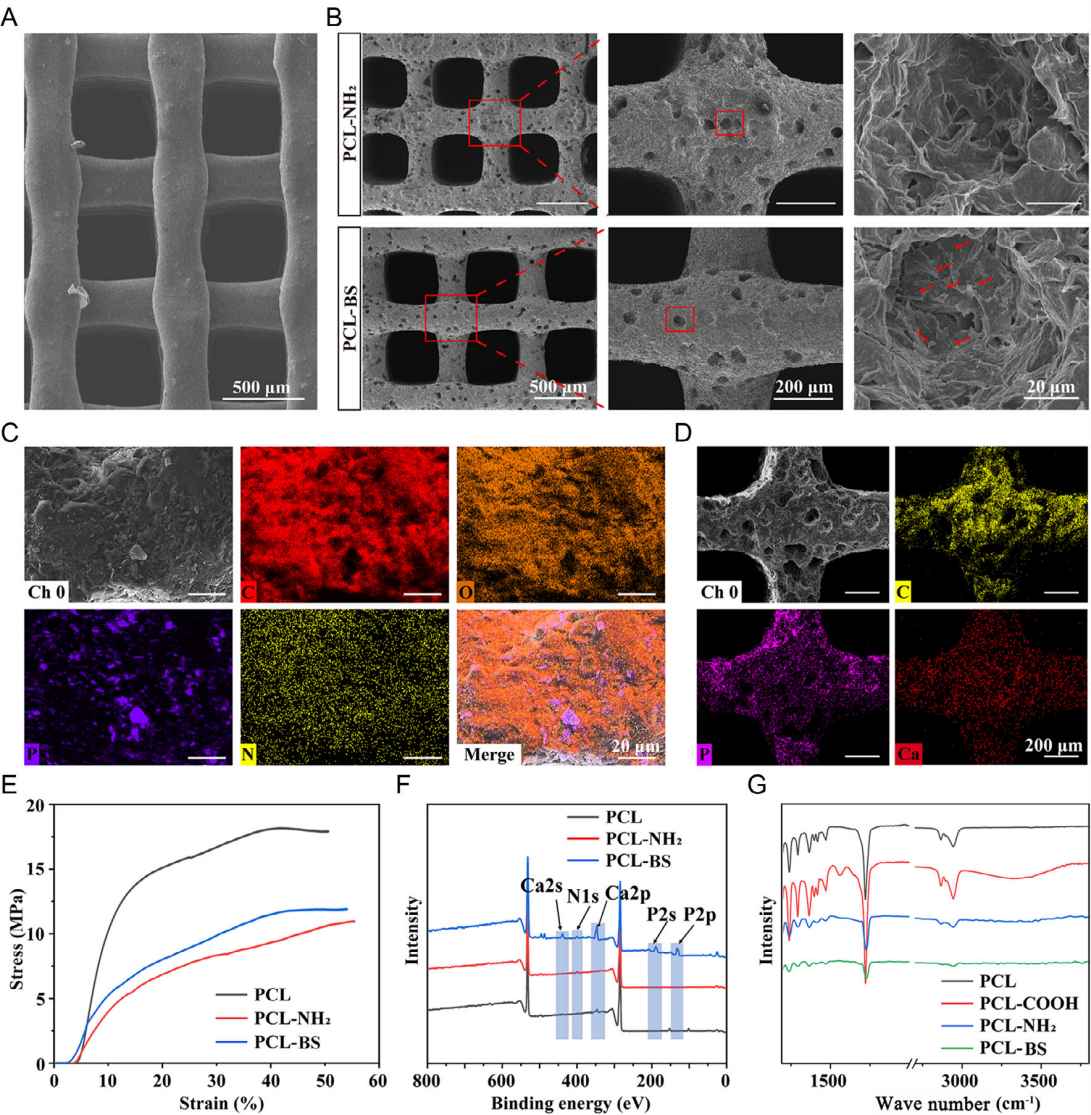
The preparation and characterization of PCL‐BS scaffolds. A) SEM images of PCL. B) SEM images of PCL‐NH_2_ and PCL‐BS scaffolds and their partial enlargement, red arrows indicate BS nanosheets. C) High‐magnification elemental mappings of PCL‐BS. D) Low‐magnification elemental mappings of PCL‐BS after mineralization in SBF buffer for 7 days. E) Compressive strength curves of PCL, PCL‐NH_2_, and PCL‐BS. F) XPS of PCL, PCL‐NH_2_, and PCL‐BS. G) FTIR of PCL, PCL‐COOH, PCL‐NH_2_, and PCL‐BS.

Next, we analyzed the microscopic morphology of PCL‐BS scaffolds by scanning electron microscopy (SEM). **Figure** **2**A,B shows SEM images of PCL, PCL‐NH_2_, and PCL‐BS scaffolds and their partial enlargement. The results illustrated that the surfaces of PCL scaffolds were relatively smooth, while the surfaces of PCL‐BS scaffolds were matted and granular. Further, local magnification demonstrated that the “BS” were evenly distributed on the surfaces (red arrow). Figure 2C shows the elemental mappings of the PCL‐BS scaffolds. Similarly, the P element (purple), representing the “BS,” existed on the surfaces of scaffolds in a sheet form, which indicated the nanosheet morphology of BS and proved the successful “seeding” of BS.

To explore the mineralization behavior of PCL‐BS scaffolds in vitro, we soaked them in simulated body fluid (SBF) solution for 7 days and then analyzed them again by elemental mappings. As shown in Figure 2D, P and Ca elements were uniformly distributed on the surfaces of the scaffolds, indicating that the PCL‐BS scaffolds could actively attract and trap Ca^2+^ in solution to form calcium phosphate mineral crystals in situ, which laid a foundation for the induction of biomineralization in vivo. In addition, bone implants should have sufficient mechanical properties to ensure reliability during bone healing. Figure 2E illustrates the compressive strength curves of scaffolds PCL, PCL‐NH_2_, and PCL‐BS. The pure PCL scaffolds exhibited the highest compressive strength at 18.3 MPa. Comparatively, the chitosan‐modified PCL‐NH_2_ scaffold demonstrated reduced strength at 10.8 MPa, while the compressive strength of the PCL‐BS scaffolds, loaded with “bone‐seeded” BS, increased to 11.5 MPa. The marginal increase in mechanical properties of PCL‐BS can be attributed to the localized nanoparticle accumulation on the scaffold surface. Notably, the PCL‐BS scaffold exhibited a lower Young's modulus (56 ± 4.28 MPa), aligning with the range of human cancellous bone (50−800 MPa), indicating promising prospects for its application as a bone tissue scaffold in advancing bone injury healing processes.

To ensure the successful “planting” of BS “BS” on PCL, we modified the scaffolds with chitosan grafting by EDC/NHS activation method to increase the adhesion of the scaffolds to BS. Fourier transform infrared spectrometer (FTIR) and XPS were used for chemical qualitative analysis of the scaffolds in each stage. Figure 2F shows the XPS profile of the scaffolds, and the results illustrated that the characteristic peak of N1s appeared at about 400 eV for PCL‐NH_2_ and PCL‐BS, proving the existence of amide bonds and indicating the successful binding to chitosan. PCL‐BS showed characteristic peaks of P2p and P2s of BP at about 182 and 264 eV, indicating that the BS was successfully inoculated on PCL. Figure 2G shows the FTIR results of the scaffolds, showing that there is a new absorption peak at 1554.94 and 3336.83 cm^−1^, which represents the stretching vibration absorption peak of —COOH and —OH in carboxylic acid, respectively. These results proved that the carboxyl group was successfully introduced into the PCL molecular chain.

### 2.3 Photothermal Effect of PCL‐BS Scaffold

**Figure 3:**
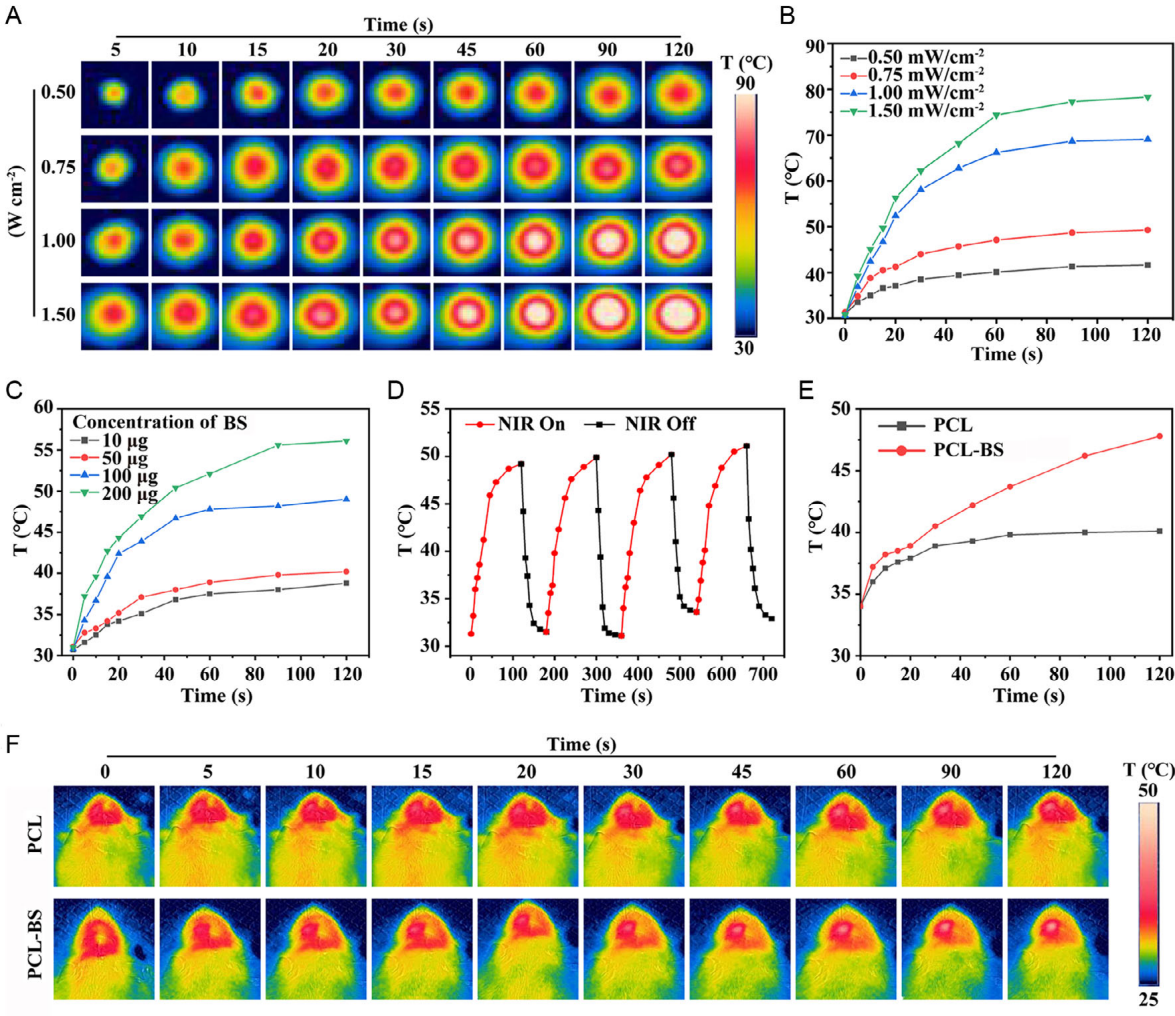
Photothermal effect of PCL‐BS. A,B) Real‐time infrared images and temperature‐varied curves of PCL‐BS scaffolds irradiated with 808 nm NIR at different light intensities (0.5, 0.75, 1.0, and 1.5 W cm^−2^) for 2 min. C) Temperature‐varied curves of PCL‐BS scaffolds irradiated with 808 nm NIR at different concentrations of BP (10, 50, 100, and 200 μg) for 2 min. D) Photothermal stability of four ON/OFF (120 s/60 s) cycles of 808 nm NIR at 1.0 W cm^−2^. E) Temperature‐varied curves of PCL‐BS and PCL scaffolds irradiated with 808 nm NIR at 1.0 W cm^−2^ for 2 min. F) Real‐time infrared images of PCL and PCL‐BS scaffolds in vivo irradiated with 808 nm NIR for 2 min.

The strong absorption from UV‐to‐NIR wavelengths endows BP with efficient photothermal conversion. Therefore, the photothermal properties of the scaffolds were investigated by NIR irradiation at various power levels (0.5, 0.75, 1.0, and 1.5 W cm^−2^) for 2 min, respectively. **Figure** **3**A,B shows the real‐time thermal imaging images and temperature of PCL‐BS scaffolds during irradiation. The results indicated that the temperature of the scaffolds was significantly improved after 2 min of irradiation with different powers; especially with the power levels of 1.0 and 1.5 W cm^−2^, the temperature reached 68 and 79 °C, respectively, which verified the exceptional photothermal conversion capability of the PCL‐BS scaffolds. In addition, considering the penetration of NIR and the thermal damage to the surrounding cells, we selected 1.0 W cm^−2^ NIR for the subsequen*t* tests. Furthermore, the photothermal properties of the PCL‐BS scaffolds were investigated by NIR irradiation with different concentrations of BP (10, 50, 100, and 200 μg) for 2 min, respectively (Figure 3C). Figure 3D shows the heating and cooling cycle curves of the scaffolds. The results showed that the change of temperature was very small even after four switching cycles (ON/OFF = 120 s/60 s), which illustrated good photothermal stability of the PCL‐BS scaffold. Figure 3E shows the temperature‐varied curves of the PCL and PCL‐BS scaffolds under the same NIR irradiation. Figure 3F shows the real‐time infrared images of PCL and PCL‐BS scaffolds in vivo. While PCL scaffolds showed minimal temperature changes, PCL‐BS scaffolds exhibited significant heating, underscoring their remarkable photothermal conversion performance.

### 2.4 Evaluation of In Vitro Biocompatibility and Osteogenic Performance

**Figure 4:**
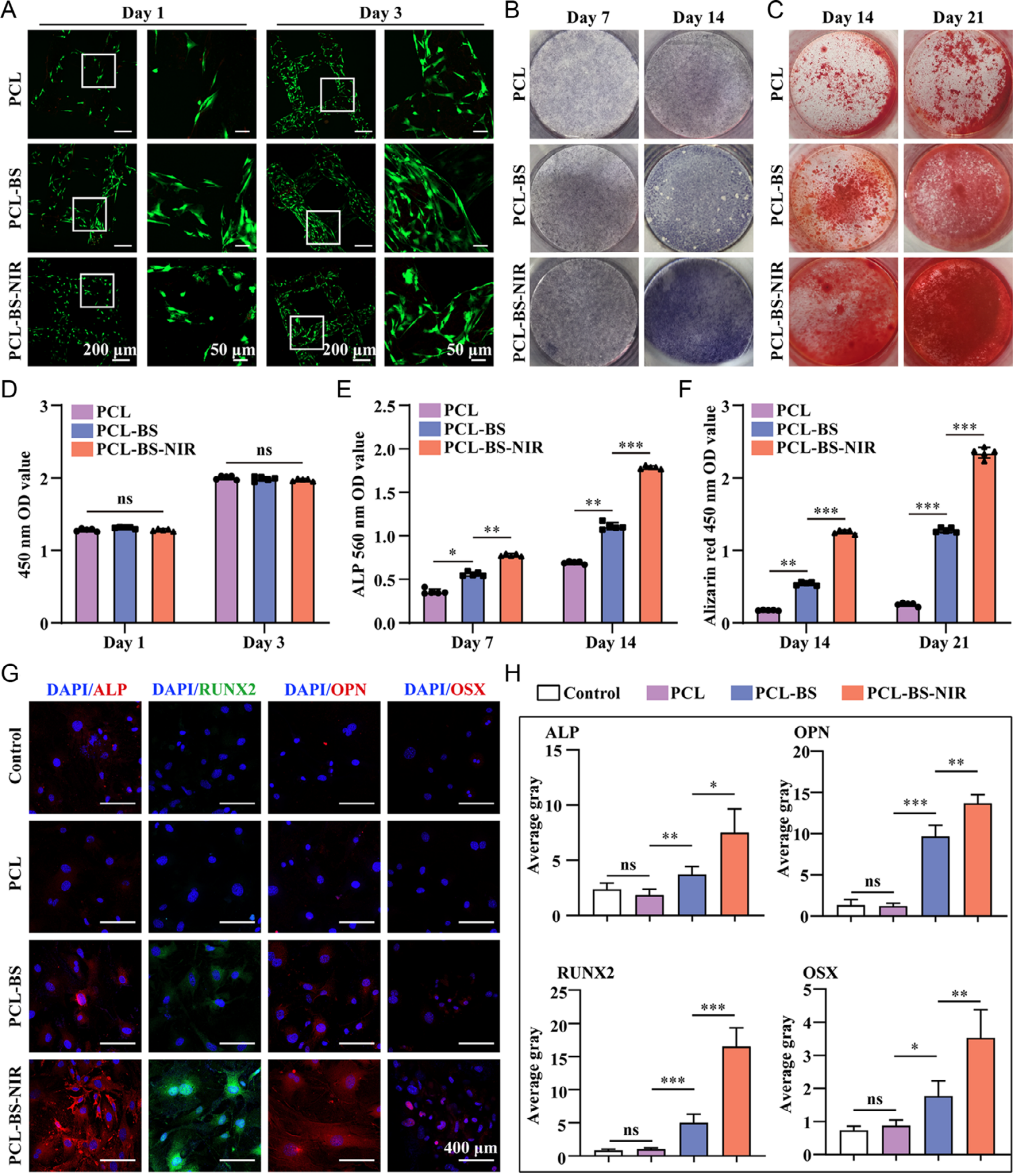
The evaluation of in vitro osteogenic performance of “BS” scaffolds. A) The live/dead staining results of BMSCs. B) The ALP staining results after 7/14 days of induction. C) The ARS staining results after 14/21 days of induction. D) The CCK‐8 results of BMSCs. E,F) The quantitative analysis of ALP staining and ARS staining on 14/21 days. G) The representative images of immunofluorescent staining of ALP, RUNX2, OPN, and OSX in BMSCs. H) The semiquantitative analysis results of fluorescence densities through software Image J.

Next, we further evaluated the biocompatibility of “BS” scaffolds. Bone marrow derived mesenchymal stem cells (BMSCs) were cocultured with the grouped scaffolds (PCL, PCL‐BS, PCL‐BS‐NIR) for 1 and 3 days to assess cell viability, adhesion, and proliferation on the scaffolds. Live/dead staining revealed robust cell survival and adherence to the scaffold surfaces across the entire culture duration. BMSCs exhibited consistent adhesion and proliferation abilities over time (**Figure** **4**A). In comparison to the control group, no notable variances in proliferation activity and cell viability were observed across all groups at different time points. Furthermore, the optical density (OD) values obtained from the Cell Count Kit 8 (CCK‐8) corroborated these findings, as depicted in Figure 4D. These results presented above confirm the excellent biocompatibility of the “BS” scaffolds and NIR irradiation without compromising cell viability. Subsequently, we further evaluated the osteogenic performance of “BS” scaffolds by detecting ALP activity, extracellular matrix (ECM) mineralization, and osteogenic‐related proteins expression levels. As an early marker, ALP has been widely recognized to reflect the level of early osteogenic differentiation.^[^ ^29^ ^]^ First, the scaffolds of PCL, PCL‐BS, and PCL‐BS‐NIR groups were cocultured with BMSCs for 7 and 14 days for ALP staining. As shown in Figure 4B, the ALP levels were notably higher in the PCL‐BS and PCL‐BS‐NIR groups compared to the PCL group. The staining density was significantly increased, with a darker color observed, particularly in the PCL‐BS‐NIR group, as validated by the absorbance at 560 nm (Figure 4E). By day 14, ALP levels were nearly 2.6 times higher in the PCL‐BS‐NIR group compared to the PCL group, demonstrating the impact of photothermal stimulation on “BS” and its role in augmenting ALP expression to facilitate bone regeneration. In addition, during the process of bone reconstruction, calcium phosphate crystals deposit in the extracellular matrix of osteoblasts, forming endogenous bone mineralization, which is crucial for promoting bone formation.^[^ ^30^ ^]^ Therefore, alizarin red staining (ARS) was further employed to stain different groups of calcium nodules to evaluate the performance of “BS” scaffolds in promoting in vitro mineralization. The staining showed that after 14 and 21 days of cocultivation, compared with the PCL group, the PCL‐BS and PCL‐BS‐NIR groups containing “BS” showed deeper staining and higher densities of mineralized nodules (Figure 4C). Subsequently, an enzyme‐linked immunosorbent assay was used for semiquantitative analysis of ARS at 450 nm. Figure 4F also confirmed that “BS” scaffolds under photothermal stimulation of NIR (PCL‐BS‐NIR group) significantly promoted calcium deposition in cocultured BMSCs. Compared with PCL group on day 14, the densities of mineralized nodules were nearly elevated by 9.3 times in the PCL‐BS‐NIR group. In summary, the “BS” scaffolds exhibited a substantial capacity to enhance calcium ion recruitment under photothermal stimulation and foster endogenous mineralized crystal formation.

Finally, to delve deeper into the osteogenic potential of “BS” scaffolds, we investigated the expression levels of pivotal osteogenic differentiation‐related proteins—ALP, runt‐related transcription factor 2 (RUNX2), osteopontin (OPN), and Osterix (OSX).^[^ ^31^, ^32^, ^33^ ^]^ RUNX2 is the main transcription factor in the regulation of osteoblast differentiation. OPN is a glycosylated protein closely related to late bone remodeling and matrix maturation. OSX is a transcription factor specific to osteoblasts and is essential for bone formation. We selected the above target proteins as osteogenic markers for immunofluorescence staining to assess the impact of “BS” scaffolds on the expression of osteogenic differentiation‐related proteins. As shown in Figure 4G, BMSCs were cocultured with PCL, PCL‐BS, and PCL‐BS‐NIR scaffolds for 7 days for immunofluorescence staining. The findings revealed minimal expression of osteogenic differentiation‐related proteins in the control group and PCL group, while the fluorescence intensities of the PCL‐BS and PCL‐BS‐NIR groups exhibited a substantial increase. The expression levels of ALP, OSX, OPN, and RUNX2 were all significantly upregulated especially in the PCL‐BS‐NIR group, demonstrating the most excellent ability of pro‐osteogenic differentiation. Subsequently, the immunofluorescence intensities of ALP, OSX, OPN, and RUNX2 were semiquantitatively analyzed by Image J software (Figure 4H). Compared with Control group, the immunofluorescence intensities of ALP, OSX, OPN, and RUNX2 were respectively elevated by 3.2, 19.5, 10.4, and 4.9 times in the PCL‐BS‐NIR group. These results showed that the “BS” scaffolds, especially when exposed to NIR photothermal stimulation (PCL‐BS‐NIR group), notably increase the production of proteins related to osteogenic differentiation in cocultured BMSCs, highlighting their crucial role in promoting osteogenic differentiation.

### 2.5 Evaluation of In Vivo Osteogenic Performance

**Figure 5:**
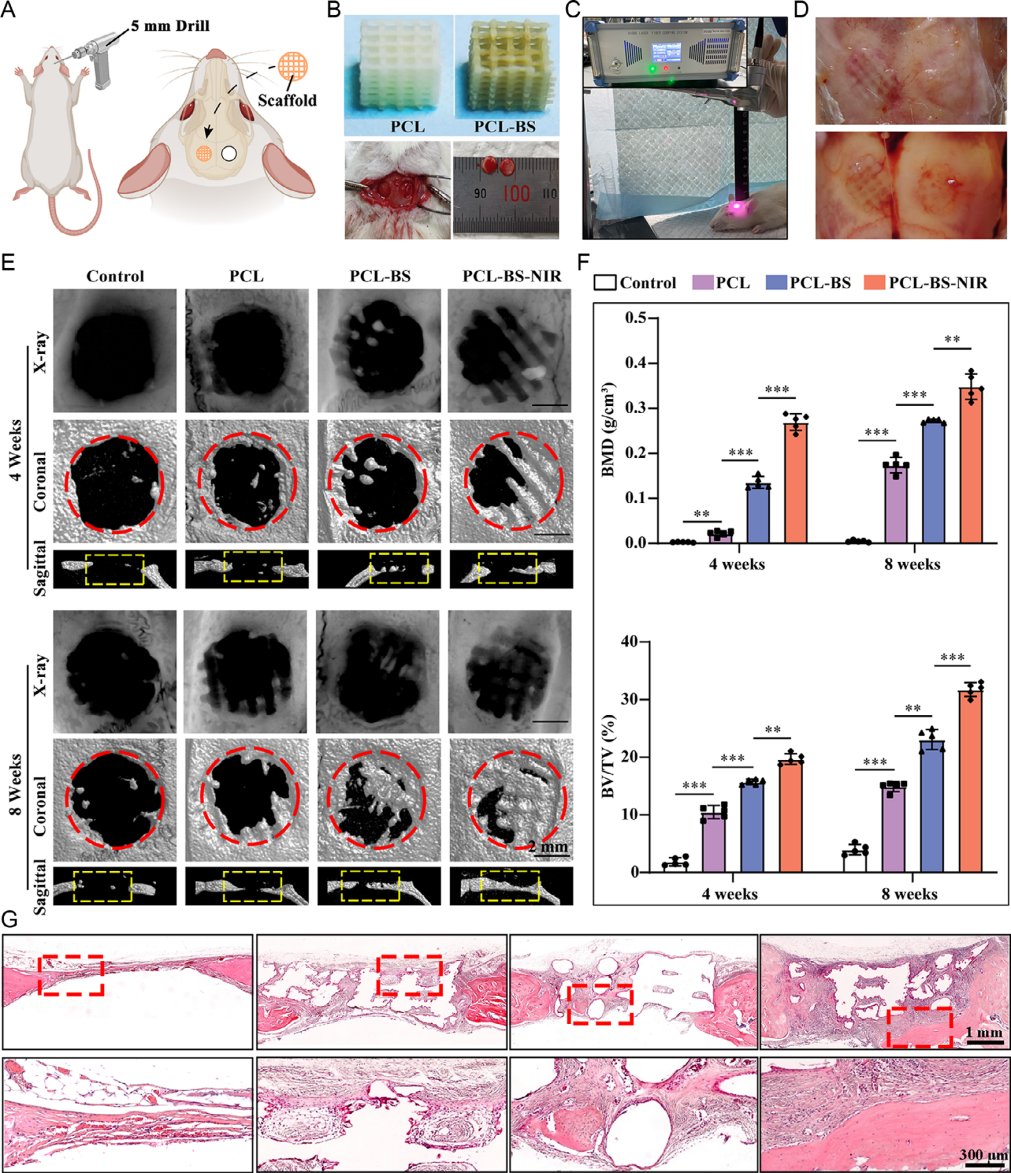
The evaluation of in vivo osteogenic performance of “BS” scaffolds. A) The diagram of animal model with skull defects of critical size. B) The macroscopic digital images of implanted scaffolds and skull defects. C) The macroscopic digital images showing the NIR radiation on skulls. D) The macroscopic digital images of skulls after regeneration of 4/8 weeks. E) The X‐ray and micro‐CT images at 4/8 weeks after the implantation of scaffolds. F) The quantitative analysis results of micro‐CT parameters. G) The histological analysis of H&E staining for the evaluation of the new bone regeneration at the site of skull defects.

An animal model with skull defects of critical size in SD rats was established (**Figure** **5**A) to evaluate the osteogenic performance of “BS” scaffolds in vivo. As illustrated in Figure 5B, two circular defects with a diameter of 5 mm were surgically made on both sides of the sagittal suture of the rat cranial parietal bone. PCL or PCL‐BS scaffolds were implanted on the one side, while no implants on the other side as control. For rats in the PCL‐BS‐NIR group, a fixed device was used to irradiate the PCL‐BS scaffolds daily with 1.0 W cm^−2^ of 808 nm NIR radiation for 120 s (Figure 5C). The skull tissues were obtained after 4 and 8 weeks (Figure 5D).

Subsequently, radiological evaluation was performed on the skull tissues, as shown in Figure 5E. X‐ray and 3D micro‐CT reconstruction (in coronal and sagittal planes) displayed the volume and structure of newly formed bone tissues in rat skull defects across various experimental groups. Notably, while 3D reconstructed images differ from X‐ray images in visual presentation, their outcomes regarding bone regeneration remain consistent. In the control group, minimal bone regeneration was noted, suggesting that a 5 mm circular bone defect cannot self‐heal without intervention. However, different levels of regenerated bone were observed in the other experimental groups. Compared with the control group, there are more regenerated bones crawling along the scaffold structure in the skull defects of the PCL group, indicating that the well‐connected network structure of the PCL scaffolds can assist in cell adhesion and proliferation, possess good bone conductivity, and serve as a bridge for bone regeneration. Over time, in all defects implanted with scaffolds, new bone formation occurred from the defect edge toward the center. Notably, the bone integration capability of the PCL‐BS scaffold surpassed that of the PCL group, indicating the “BS” modification's potential to enhance bone induction. This modification fosters cell migration, recruitment, development, differentiation, and osteogenesis. During an 8‐week healing cycle, the PCL‐BS‐NIR group achieved the best level of skull defect repair, with regenerated bone almost filling the entire defect area for complete healing. This suggests that the local photothermal stimulation of NIR effectively contributed to promoting new bone regeneration. Figure 5F presents the assessment of bone volume/tissue volume ratio (BV/TV) and bone mineral density (BMD) across all groups. The results showed that the BV/TV of PCL‐BS‐NIR group (19.69 ± 0.93% and 31.76 ± 1.21% respectively at weeks of 4 and 8) was nearly 10 times higher than the Control group (1.89 ± 0.69% and 3.96 ± 0.90% respectively at weeks of 4 and 8). Similar results were also shown in BMD analysis, where the average values were 0.269 ± 0.018 and 0.348 ± 0.028 g cm^−3^ respectively at weeks of 4 and 8 in the PCL‐BS‐NIR group, which are much higher than the Control group (0.002 ± 0.001 and 0.005 ± 0.002 g cm^−3^ at weeks of 4 and 8, respectively). The PCL‐BS‐NIR group demonstrated significantly superior bone regeneration induction compared to the other groups.

Additionally, the histological analysis of hematoxylin and eosin (H&E) staining further assessed the new bone regeneration at the skull defect site (Figure 5G). Notably, in the control group, the bone defect areas were solely occupied by soft tissues, with negligible signs of new bone formation. In contrast, minimal new bone formation is visible at the periphery of the bone defects in the PCL group. However, in the PCL‐BS group, particularly in the PCL‐BS‐NIR group, there is noticeable growth of new bone extending from the periphery to the center of the defect. In addition, collagen type I (COL‐I) plays a pivotal role in bone ECM formation and the linkage between cell surface receptors and ECM proteins. Runt‐related transcription factor 2 (RUNX2) is a key osteogenic gene involved in bone formation and exerts a crucial influence on regulating osteogenic differentiation. Therefore, we selected these two target proteins as markers and used immunofluorescence staining to further investigate the expression of proteins related to osteogenic differentiation. As depicted in Figure S1, Supporting Information, Col I and RUNX2 exhibited minimal expression around the scaffolds in both control and PCL groups, as evidenced by their weak fluorescence intensity. However, for the PCL‐BS‐NIR group, there was significantly enhanced fluorescence intensity observed for Col I and RUNX2 protein expression surrounding its scaffold area. This result can be attributed to the high absorption of BS in the NIR region, which under NIR light facilitates the degradation of BS to form free phosphate and the adsorption of surrounding Ca^2+^ to promote endogenous mineralization.^[^ ^34^ ^]^ Good photothermal conversion ability of BS can generate local mild thermal stimulation, which further promotes the upregulation of ALP, HSP, and bone morphogenetic protein, accelerating the process of bone repair.^[^ ^35^ ^]^ In summary, the “BS” scaffolds under NIR photothermal stimulation, namely, the PCL‐BS‐NIR scaffolds, have the most excellent abilities of bone conduction, bone induction, and bone integration, which can effectively promote new bone regeneration.

### 2.6 The Transcriptome Mechanism of BS Involvement in Regulating Signal Transduction and Network Regulation in Bone Regeneration

**Figure 6:**
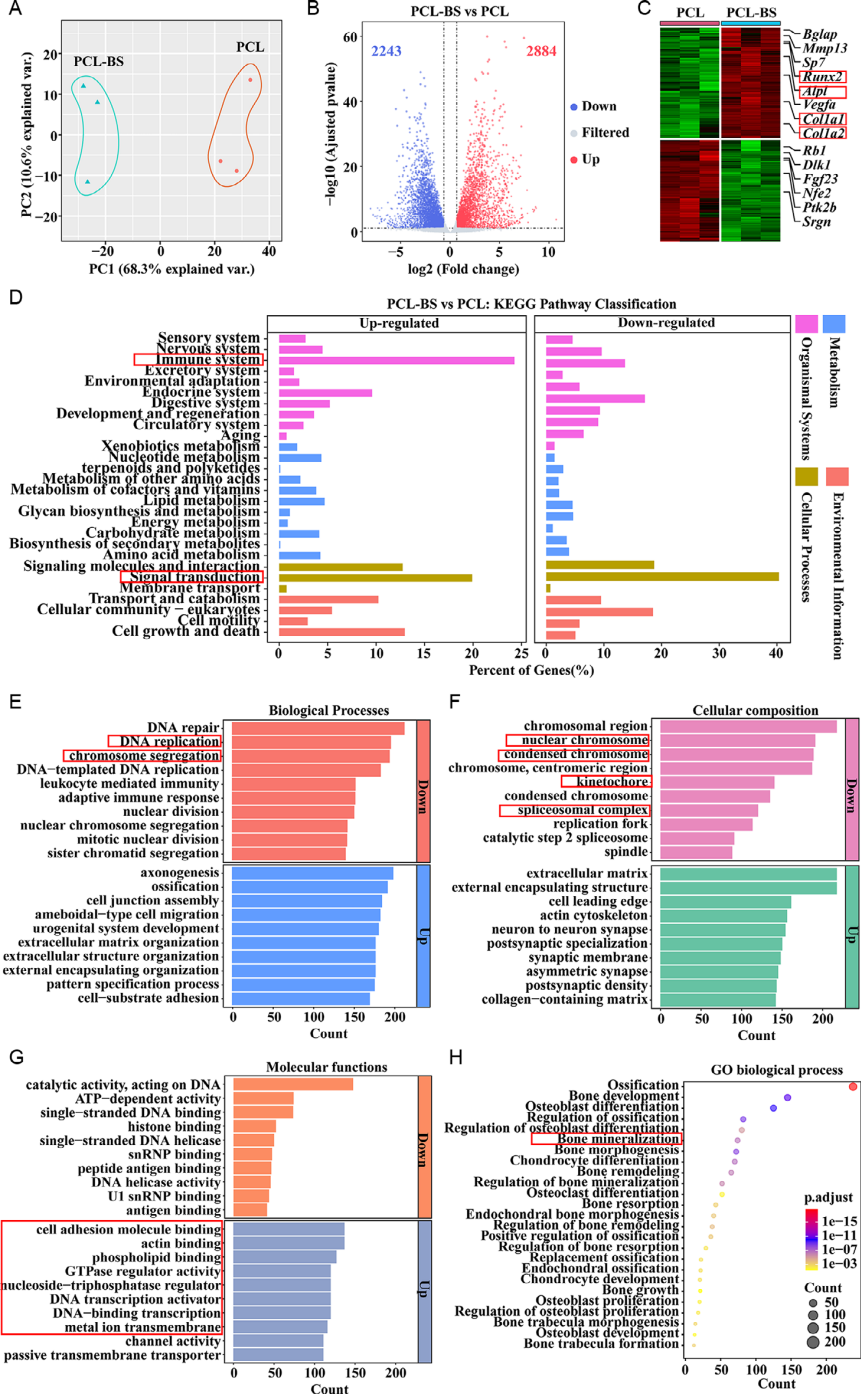
Differential gene expression between PCL‐BS and PCL groups was analyzed by RNA‐seq 4 weeks after scaffold implantation into the bone defect. A) PCA analysis of all samples. B) The DEGs between the PCL‐BS and PCL group. C) Heatmap analysis of DEGs. D) KEGG enrichment analysis of DEGs between PCL‐BS and PCL group. E–G) The top 30 up enrichment GO terms in the PCL‐BS group compared to the PCL group. H) Up enrichment GO terms related to the bone in the PCL‐BS group compared to the PCL group.

To elucidate the mechanism underlying BS's impact on gene expression and signal transduction during bone regeneration, we removed the scaffolds and tissue cells that grew into the scaffolds 4 weeks after implantation and then performed full transcriptome sequencing (RNA seq) analysis. The transcriptome data was validated for further analyses through principal component analysis (PCA) (**Figure** **6**A). In comparison to the PCL group, the PCL‐BS group exhibited 2884 upregulated differentially expressed genes (DEGs) (>1.6‐fold, *p* < 0.05) and 2243 downregulated DEGs (<0.65‐fold, *p* < 0.05) (Figure 6B). The heatmap displays the expression patterns of DEGs (Figure 6C). In line with previous findings,^[^ ^36^, ^37^ ^]^ several key genes associated with osteogenic differentiation, such as COL‐1, RUNX2, and ALP, were found to be highly expressed in the PCL‐BS group.

In order to gain a deeper insight into the metabolic pathways affected by BS, we employed Kyoto Encyclopedia of Genes and Genomes (KEGG) enrichment analysis to uncover the associated functions of DEGs. The analysis revealed that signal transduction‐related genes accounted for the highest proportion among both the upregulated and downregulated genes (Figure 6D). This observation aligns with the intricate protein synthesis and cellular communication inherent in bone regeneration processes. Among the upregulated genes, the proportion of genes related to the immune system is high, in line with previous findings.^[^ ^38^, ^39^ ^]^ BS has great potential for immune regulation. However, the exact function of BS‐mediated immune processes in bone regeneration is still uncertain, and it is known that inflammation is crucial for bone regeneration,^[^ ^40^ ^]^ which may be another important mechanism for BS to accelerate bone regeneration.

GO enrichment analysis was utilized to delve into the functional roles of DEGs across biological processes, cellular components, and molecular functions, focusing on the top 30 terms with the highest enrichment compared to the PCL group. In terms of downregulation, biological processes are mainly related to cell mitosis, including biological processes related to DNA replication and chromosome separation (Figure 6E). The main cellular components involved in this biological process include nuclear chromosomes, kinetochore, condensed chromatin, and spliceosome complexes (Figure 6F). The molecular function reveals that the downregulation of genes in inhibiting mitosis mainly affects ATPase activity, histone binding, DNA helicase activity, and snRNP binding in DNA (Figure 6G). This is consistent with previous reports that BS can inhibit cell proliferation and has antitumor potential.^[^ ^39^ ^]^ In terms of upregulation, biological processes are mainly related to bone mineralization, cell junctions, and extracellular matrix composition (Figure 6E). The main cellular components involved in this biological process include extracellular matrix, external packaging structure, cytoskeleton, and synapses between neurons (Figure 6F). The molecular function reveals that upregulation of genes mainly affects adhesion molecule binding, actin binding, phospholipid binding, GTP enzyme regulatory activity, DNA binding transcriptional activator activity, and ion channel activity (Figure 6G). To further elucidate how BS affects bone regeneration, we conducted GO enrichment analysis on bone‐related terms and found that BS mainly affects the mineralization process, followed by the differentiation and proliferation of osteoblasts, osteoclasts, and chondrocytes (Figure 6H). These findings collectively underscore BS's multifaceted role in inhibiting mitosis, promoting biomineralization, influencing extracellular matrix generation, and providing deeper insights into its mechanisms in bone regeneration.

## 3 Conclusion

With inspiration from natural processes of seeding, flowering, and fruit bearing, a “BS” scaffold (PCL‐BS) was prepared by pretreatment of BP and subsequent conjugation with chitosan‐modified PCL scaffold. The results of in vitro experiments showed that the “BS” uniformly distributed on the surface of the PCL scaffolds, actively captured the surrounding Ca^2+^, and then formed calcium phosphate mineral crystals in situ. In vitro, cell experiments showed that the PCL‐BS scaffolds significantly promoted the mineralization behavior of osteoblasts under 808 nm NIR irradiation. In addition, we further demonstrated the excellent osteogenic properties of the PCL‐BS scaffolds through in vivo experiments with critical bone defects in the rat skull. After 4 weeks of scaffold implantation, the cells and tissues that grew into the scaffolds were removed and analyzed by RNA‐seq analysis, which illustrated that BS could inhibit mitosis and promote biomineralization and extracellular matrix formation. Overall, PCL‐BS scaffolds were suitable for bone tissue regeneration by capturing calcium ions to accelerate the endogenous mineralization.

## 4 Experimental Section

4.1

4.1.1

##### Preparation of BP “Bone Seeds”

BP nanosheets were produced as described in previous studies.^[^ ^10^, ^40^ ^]^ Briefly, 30 mg BP powder was dissolved in an ice bath and subjected to a 12 h 300 W sonication treatment in a 30 mL NMP solution, using equipment from Ningbo Scientz Biotechnology, China. The resulting gray suspension underwent a 10 min centrifugation at 4000 rpm to eliminate any remaining unexfoliated BP particles. The supernatant, containing exfoliated BP nanosheets, was collected and subjected to a reaction with varying concentrations of ammonium hydroxide (1, 5, 10, 15, 25 mM) while continuously stirring at 40 °C for 2 h to produce “BS”. Subsequently, the BS were centrifuged at 15 000 rpm, resuspended in the NMP solution, and stored at four degrees in the dark for future applications.

##### Capturing Calcium Ions by BS

To verify the ability of BS to recruit calcium ions, first, the weakly alkali‐treated BP was centrifuged at 12 000 rpm (200 μg mL^−1^), 1 mL of 0.1 m CaCl_2_ solution was gradually added, and incubated for 12 h at room temperature with continuous stirring in darkness. After centrifugation at 12 000 rpm, the supernatant was collected to detect the concentration of calcium ions by Calcium Assay Kit (Beyotime, China). Subsequently, washing steps with ethanol and water were performed to eliminate any unabsorbed calcium ions.

##### Fabrication of 3D “Bone Seeds” Scaffold

PCL was bought from Sigma with the molecular weight of 80 000. The design of the 3D scaffold incorporated square pores and orthogonal cubic lattice disks. The overall dimensions of the scaffolds were 30 mm × 30 mm × 5 mm while the wire diameter was 200 μm, and the size of square pores was 400 μm. After fabrication, for further analysis, the fabricated scaffolds were delicately sectioned into smaller pieces, ensuring minimal architectural damage.

The PCL scaffolds underwent immersion in a 5 M sodium hydroxide (NaOH) solution at 37 °C with a shaking speed of 60 rpm for 24 h. Subsequently, thorough rinsing with water for 1 h was performed to eliminate any remaining NaOH. The scaffolds were then dried in a vacuum oven at 30 °C, resulting in the fabrication of porous carboxyl‐functional scaffolds, referred to as PCL‐COOH. These scaffolds were further treated by immersion in 10 mL MES buffer (10.0 mM, pH = 5.5), containing 1‐ethyl‐3‐(3‐dimethylaminopropyl)‐carbodiimide hydrochloride (EDC, 120 mg) and N‐hydroxysuccinimide (NHS, 180 mg) for 2 h to activate the carboxylic groups of 3D PCL scaffold. Then these scaffolds were rinsed with water for three times and immersed in chitosan solution (0.5 mg mL^−1^) for 24 h to graft with amino groups of chitosan. PBS‐washed, nitrogen dried, and the amino‐modified PCL scaffold was obtained (PCL‐NH_2_). Finally, these PCL‐NH_2_ scaffolds were soaked in BS solution to plant seeds onto the porous scaffolds. After being rinsed with double distilled water three times, the PCL‐BS scaffolds were fabricated successfully.

##### Characterization of BS and PCL‐BS Scaffolds

TEM (TALOS F200X) at 200 kV voltage was used to observe the morphology of BP nanosheets and BS. Raman spectroscopy and XPS were used to investigate the capture of Ca^2+^. DLS was used to analyze the zeta potential and particle size distribution between BP and BS. For assessing the macropores and microstructure of PCL and PCL “BS” scaffolds, SEM (Sirion 2000) was utilized. The amount and distribution of planted “BS” were characterized by energy‐dispersive Xray spectrometry element mapping. FTIR and XPS were employed to verify the successful plant of “BS” and mineralization. The scaffolds underwent compression analysis along the vertical (*z*‐axis) using a universal testing machine (Instron5569) operating under controlled displacement (5 mm min^−1^). As for the releasing of PO_4_^3−^ and Ca^2+^ from PCL, “BS” scaffolds were measured by ion chromatography (iCAP7600). In addition, the mineralization capacity of PCL‐BS in vitro was soaked in SBF solution and assessed after 7 days for soaking.

##### Photothermal Conversion Capability of PCL‐BS Scaffolds

The photothermal conversion capability of “BS” scaffold (10, 50, 100, 200 μg mL^−1^) was assessed under NIR (808 nm, FCR5, BOHR Electronic Technology Co., China) light with varying power densities (0.5, 0.75, 1.0, and 1.5 W cm^−2^) and different times and was recorded by the infrared thermal imaging camera (FLIR, USA) as real‐time thermal imaging and the maximum temperature. To test the stability of photothermal in vitro, “BS” scaffold was exposed to NIR at 1.0 W cm^−2^ for 120 s (laser on), then cooled down for 60 s (laser off), and NIR on/off cycle was repeated for four times. Furthermore, to test the stability of photothermal in vivo, PCL and “BS” scaffolds were implanted in critical size rat calvarial defect model and exposed to the 808 nm irradiation at 1.0 W cm^−2^ for 120 s.

##### Cell Viability and Proliferation Assay

Using methods described in previous studies,^[^ ^41^ ^]^ live/dead staining was assessed by a live/dead kit (Invitrogen, US), where green meant viable labeled by Calcein‐AM and red suggested cell death stained by ethidium homodimer‐1. After 1 and 3 days of culture, cells on the scaffolds were stained with working solution for 30 min and observed by laser confocal fluorescence microscope (Zeiss, Germany). To evaluate the cell proliferation on different scaffolds, BMSC cells were seeded in 96‐well plates at a density of 2 × 10^4^ well^−1^ and incubated with the leach liquor of scaffolds. Following incubation for 1 and 3 days, CCK‐8 solution was introduced into individual wells and incubated for an additional 4 h at 37 °C. Then, cell viability was assessed at 450 nm.

##### ALP Staining and Alizarin Red s Staining

BMSCs were cocultured with the functioned scaffolds and cells cultured with PCL scaffolds were assigned as control group. Upon reaching 80% density, the osteoinductive medium (Cyagen, China) replaced the standard DMEM medium for cells differentiation culture. On days 7 and 14 of the induction period, the presence of ALP was visualized through staining as per the specified protocol (utilizing the ALP Color Development Kit from Beyotime, China), and the outcomes were captured using a digital camera. ALP activity was assessed at a wavelength of 560 nm. Cells were subjected to ARS staining (ARS, Cyagen, China) to visualize calcium nodules at day 14 and 21. While to quantify the calcium nodules stained by ARS, 10% acetic acid was added to the wells. Subsequent to an overnight incubation, the solution's OD was assessed at a 450 nm.

##### Immunofluorescence Staining

Rat BMSCs were cultivated in both regular and osteoinductive media supplemented with PCL, PCL‐BS scaffolds, and PCL‐BS scaffolds‐NIR as described above. At day 7, cell fixation was carried out utilizing a 4% paraformaldehyde solution, followed by PBS washing and subsequent storage at 4 °C until cytochemistry labeling. Permeabilization of cells was achieved through a 15 min treatment with 0.2% v/v Triton X‐100, while nonspecific binding was blocked using a 10% solution of goat serum (Invitrogen, US). Following these procedures, they were incubated overnight at 4 °C with the primary antibodies: Osteopontin (ab8448, Abcam, 1:200), Osterix (ab22552, Abcam, 1:200), ALP (NB110‐3638, Novus, 1:200), and RUNX2 (ab236639, Abcam, 1:200). Subsequent to the primary antibody incubation, cells underwent PBS washing and were then exposed to suitable Alexa Fluor‐coupled secondary antibodies (Molecular Probes, Life Tech, US, 1:400) for 1 h. The nuclei were stained using DAPI, followed by extensive PBS washing, preceding the qualitative analysis conducted using laser scanning confocal microscopy (LSCM).

##### Rat Calvarial Critical Size Defect Model

The experimental methods, housing, and care of the animals were approved and conducted in accordance with the animal experimentation regulations of the Animal Ethics Committee of Shanghai Jiaotong University (SYXK (Hu) 2018‐0027). 2% pentobarbital sodium was performed to anesthetize SD rats through intraperitoneal injection. After shaving the fur and sterilization, a longitudinal incision was performed in the middle of the head to expose the calvarium. Two bilateral full‐thickness defects of 5 mm were created using a dental trephine. The rats were divided into four groups (*n* = 6): control, PCL, PCL‐BS, and PCL‐BS‐NIR: PCL‐BS with 808 nm NIR irradiation (1.0 W cm^−2^, 60 s day^−1^, day 1–7). Penicillin was injected once a day postoperatively for 3 days.

##### Radiography Analysis

The X‐ray machine was used to assess the in vivo bone regeneration of implanted scaffolds in the calvarial specimens which harvested at 4 and 8 weeks of operation. Furthermore, Micro‐CT (SCANCO, Switzerland) was further used to evaluate the regenerative condition within the defect areas at a spatial resolution of 15 μm. The SCANCO software was utilized to reconstruct the 3D calvarium structures. Cylindrical shapes measuring 5 mm in diameter and approximately 1 mm in height were chosen to encompass the defect regions as the volume of interest (VOI). Quantitative analysis of bone formation within these VOIs involved the calculation of BMD and BV/TV.

##### Histological Analysis

The harvested craniums were fixed in 4% paraformaldehyde for 48 h, decalcified with 10% EDTA (Sigma‐Aldrich) for 4 weeks, followed by embedding in paraffin and sectioning into 5 μm thick for H&E histological analysis to evaluate the regeneration condition.

##### The Whole Transcriptome Analysis

RNA extraction, mRNA library preparation, and sequencing procedures were executed following established methodologies as previously described.^[^ ^42^ ^]^ Subsequent to the compilation of the final transcriptome, String‐Tie and ballgown algorithms were employed to gauge the expression levels of transcripts and genes by computing FPKM (FPKM = [total_exon_fragments/mapped_reads (million) × exon_length (kB)]). Differential expression analysis was conducted using the R package edgeR, selecting transcripts and genes exhibiting fold changes >1.5 or <0.66, with a significance threshold of *P* < 0.05.

##### Statistical Analysis

The data were presented as means ± standard deviations. Statistical analyses (GraphPad Software, USA) primarily employed one‐way ANOVA followed by Tukey's multiple comparison test for intergroup variance assessment, unless specified otherwise. *P* < 0.05 indicated statistical significance.

## Conflict of Interest

The authors declare no conflict of interest.

## Supporting information

Supplementary Material

## Data Availability

The data that support the findings of this study are available from the corresponding author upon reasonable request.
