# Supplementary material for: Bioinspired Bone Seed 3D‐Printed Scaffold via Trapping Black Phosphorus Nanosheet for Bone Regeneration
Source: Small Sci. 2024 May 11;4(6):2300357. doi: 10.1002/smsc.202300357 (PMC11935154; doi:10.1002/smsc.202300357)
Supplement: Supplementary file 1 — Supplementary Material [file SMSC-4-2300357-s001.pdf]

supporting information

Bioinspired bone seed 3D printed scaffold *via* trapping black phosphorus nanosheet for bone regeneration

Zhengwei Cai, Zhijie Chen, Yuan Tang, Liang Cheng, Minglong Qiu, Ningtao Wang, Wei Jiang, Zhanchun Li, Yunhai Zhang*, Bruno Sarmento*, Wenguo Cui*

Z. Cai, Z. Chen, Y. Tang, M. Qiu, N. Wang, Y. Zhang, W. Cui.
Department of Orthopaedics, Shanghai Key Laboratory for Prevention and Treatment of Bone and Joint Diseases, Shanghai Institute of Traumatology and Orthopaedics, Ruijin Hospital, Shanghai Jiao Tong University School of Medicine, 197 Ruijin 2nd Road, Shanghai 200025, P. R. China.
E-mail addresses: yunhai_shanghai@163.com (Y. Zhang), wgcui80@hotmail.com (W. Cui).
B. Sarmento.
I3-Instituto de Investigação e Inovação Em Saúde and INEB-Instituto de Engenharia Biomédica, Universidade Do Porto, Rua Alfredo Allen 208, 4200-135, Porto, Portugal; IUCS-Instituto Universitário de Ciências da Saúde, CESPU, Rua Central de Gandra 1317, 4585-116, Gandra, Portugal.
E-mail addresses: bruno.sarmento@i3s.up.pt (B. Sarmento).
Z. Chen, L. Cheng, Z. Li.
Department of Orthopaedic Surgery, Renji Hospital, School of Medicine, Shanghai Jiao Tong University, Shanghai 200127, P. R. China.
Y. Zhang.
Department of Orthopedics, Wuxi Branch of Ruijin Hospital, Wuxi, Jiangsu 214106, P. R. China.
Wei Jiang
Department of Orthopedics, The First Affiliated Hospital of Anhui Medical University, 218 Jixi Road, Shushan District, Hefei, Anhui, 230022, PR China.

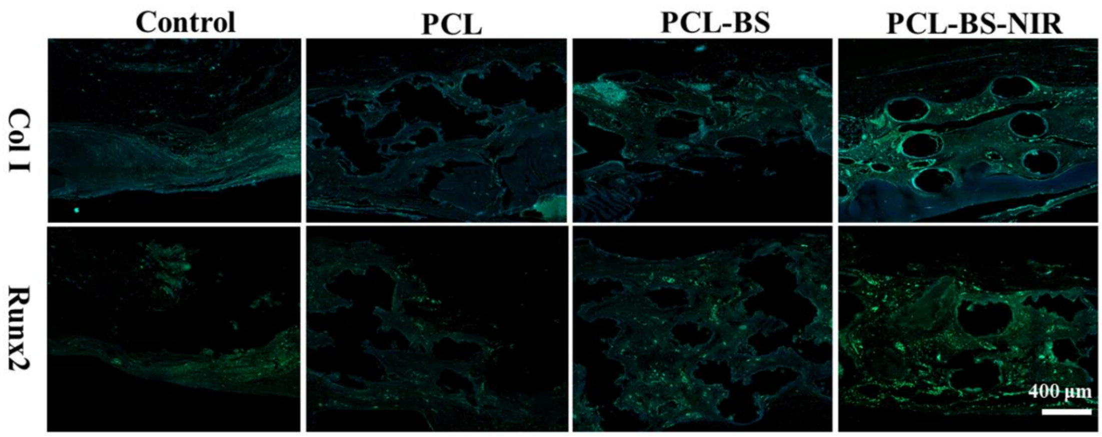

Figure S1. The immunofluorescence images of Col I and Runx2 expression in new formed tissue *in vivo*.
